# Clinical and pathological characteristics of patients with colorectal cancer under age stratification

**DOI:** 10.3389/fonc.2025.1656277

**Published:** 2026-01-12

**Authors:** Zhuoran Liu, Wei Kong, Yingmin Lin, Tengfei Yin, Min Wang

**Affiliations:** 1 Department of Gastroenterology, Qilu Hospital of Shandong University, Jinan, Shandong, China; 2 Cheeloo College of Medicine, Shandong University, Jinan, Shandong, China; 3 Department of General Practice, Qilu Hospital of Shandong University, Jinan, Shandong, China; 4 Department of Geriatric Medicine, Gastroenterology, Qilu Hospital of Shandong University, Jinan, Shandong, China; 5 Laboratory of Gerontology and Anti-Aging Research, Qilu Hospital of Shandong University, Jinan, Shandong, China

**Keywords:** age stratification, clinical manifestations, colorectal cancer, early-onset colorectal cancer, pathological characteristics

## Abstract

**Background:**

The incidence of early-onset colorectal cancer is increasing. The rate of early diagnosis and screening is low, and the prognosis is poor. This study aims to compare the clinical and pathological characteristics of colorectal cancer patients under age stratification, so as to improve the awareness of prevention in different populations and provide the basis for treatment strategies.

**Methods:**

This retrospective cross-sectional study included patients who underwent electronic colonoscopy and were diagnosed with colorectal cancer by pathology in Qilu Hospital of Shandong University from July 2017 to June 2020. Their clinical and pathological data were statistically analyzed according to <40 years, 40–50 years and >50 years groups.

**Results:**

850 patients were included (40 <40 years, 108 40–50 years, and 702 >50 years). The proportions of comorbidities (7.5% vs 26.8% vs 55.4%, p=0.006) and medical history were higher (12.5% vs 11.1% vs 17.1%, p=0.163) in the >50 years group; the proportion of family history was higher in the <40 years group (10.0% vs 9.3% vs 3.8%, p=0.015). All patients in the age group under 40 years had symptoms (100% vs 96.3% vs 93.4%, p=0.161), while some patients in the other two groups did not. The incidence of left-sided colon was higher in the <40 years group (37.5% vs 25.0% vs 23.1%, p=0.392); the incidence of right-sided colon was higher in the 40–50 and >50 years group (10.0% vs 25.9% vs 21.9%, p=0.392). Low-grade (7.5% vs 5.6% vs 5.4%, p=0.057), mucinous adenocarcinoma (15.0% vs 9.3% vs 3.6%, p=0.002) and signet ring cell carcinoma (5.0% vs 0.9% vs 0.3%, p=0.002) were more common in the <40 years group. Late-stage tumors were more common in the <50 years group (65.0% vs 51.9% vs 46.6%, p<0.01); early-stage tumors were more common in the >50 years group (35.0% vs 48.2% vs 53.4%, p<0.01).

**Conclusion:**

There are more family history in early-onset colorectal cancer patients, with various symptoms, mucinous adenocarcinoma and signet ring cell carcinoma, poor tumor differentiation and late stage. Young adults with symptoms should undergo colonoscopy actively. For patients <40 years, genetic testing should be performed.

## Introduction

Colorectal cancer (CRC) is a highly prevalent malignant tumor in the digestive system, ranking third globally in incidence and second in mortality (1). In China, CRC ranks fourth in both incidence and mortality (2). In recent years, the incidence of early-onset colorectal cancer (EOCRC) has been on the rise, attracting significant attention (3, 4). EOCRC typically refers to CRC diagnosed before the age of 50 (5). Compared to late-onset colorectal cancer (LOCRC) diagnosed after age 50, EOCRC exhibits unique clinical and pathological features, with significant differences in its pathogenesis, risk factors, and prognosis. Risk factors for CRC include late-stage age, male gender, family history, inflammatory bowel disease, diabetes, obesity, consumption of red meat and processed meats, smoking, heavy drinking, and a high-fat and low-fiber diet (6). Research has shown that Helicobacter pylori infection is an independent risk factor for CRC (7), and our team has also conducted related studies (8).

CRC is more common in people over 50 years old, but epidemiological studies at home and abroad have confirmed that CRC is showing a trend of gradually affecting younger people. According to statistics, among the cancer burden of Adolescents and Young Adults (AYA) worldwide, the incidence of CRC has recently shown an increasing trend (9). A cross-national cohort study confirmed that the incidence of EOCRC has generally increased across five continents worldwide, with the United States, Canada, Australia and other countries being particularly prominent (10). A controlled study covering 50 countries found that the incidence of EOCRC was on the rise in 27 countries (27/50), among which the rate of increase in the incidence of EOCRC in 20 countries exceeded that of LOCRC (20/27) (11). An epidemiological survey covering 20 European countries revealed that the incidence of EOCRC rose significantly in 70% (14/20) of the countries, remained stable in 25% (5/20), and only Italy showed a downward trend (12). According to statistics from the American Cancer Society (ACS), approximately one in every ten new CRC patients is under 50 years old, accounting for 10% to 12% of all newly diagnosed cases (10). The situation in China is equally severe. Zhang et al. found that the prevalence of CRC in China increased to 2.66 times from 1990 to 2017. Among them, the group with the most significant increase was the 15–49 age group, with the prevalence increasing to 2.4 times (13).

Many studies have shown that common clinical manifestations of EOCRC include abdominal pain, rectal bleeding, weight loss, anemia, decreased appetite, altered bowel habits and intestinal obstruction symptoms (14). In terms of anatomical distribution, EOCRC lesions are predominantly located in the left colon, particularly in the rectum and sigmoid colon (15). Research by VUIK et al. revealed that compared to LOCRC, EOCRC exhibits more aggressive pathological features: higher prevalence of signet ring cell carcinoma, generally poorer tumor differentiation, more frequent stage III disease, and increased lymph node metastasis risk (16). This is similar to the results of a large-scale cohort study in China (17), which found that compared with LOCRC, the EOCRC group had fewer comorbidities, more symptom manifestations, and a higher proportion of rectal cancer. Tumors are more aggressive, with a higher incidence of poorly differentiated, mucinous adenocarcinoma and signet ring cell carcinoma, and the advanced stage of tumors is later. Some studies have also found that EOCRC patients show significantly higher incidence of metastatic disease at initial diagnosis compared to LOCRC controls (18); synchronous or metachronous tumors are also more common in EOCRC (19). While most previous CRC studies focused on patients aged 50 and older, our research advances the age range to 40, which represents an innovative aspect of this study.

Previously, both domestically and internationally, the target age for CRC screening was generally set between 50 and 75 years old. Currently, the recommended screening age has been moved forward. The American Cancer Society (ACS) and the American College of Gastroenterology (ACG) recommend that CRC screening should begin at age 45 for the general population (20, 21). The CRC screening guidelines released by China (22) state that, considering the actual national conditions that the incidence of CRC in the Chinese population begins to rise from the age of 40, it is recommended that the general population start to undergo risk assessment for CRC from the age of 40, and that high-risk groups with assessment results receive screening for CRC between the ages of 40 and 75. Most existing studies set the age limit for EOCRC at < 50 years old, and the research data for the 40-year-old age group provide insufficient support. In addition, people aged 40 to 50 may represent a transitional stage from hereditary to sporadic and from early-onset to late-onset, and their tumor characteristics may combine the features of both. Therefore, we further grouped EOCRC with the age of 40 as the boundary to explore the potential differences in genetic background, clinical characteristics, and tumor biological behavior between people under 40 years old and those aged 40-50.

To sum up, the incidence of EOCRC is on the rise globally, with the age of onset gradually getting younger and the screening age advancing. Most existing studies compare EOCRC with LOCRC, and research on younger CRC patients is relatively limited. Therefore, in this study, EOCRC patients were divided into three groups: <40 years old, 40–50 years old, and >50 years old, aiming to explore the differences in CRC characteristics under a more refined age stratification. This move conforms to the current situation at home and abroad where the age for CRC screening is advanced, enriches the previous research content of our team on CRC (23–30), and provides strong support for promoting the “Healthy China” strategy. By systematically comparing the differences in clinical and pathological characteristics of 850 CRC patients (< 40 years old, 40–50 years old and > 50 years old), we aim to deepen our understanding of the characteristics of CRC in different age groups and provide a basis for establishing an age-specific screening system and prevention and treatment strategies.

## Materials and methods

### Study design

This was a retrospective cross-sectional study that analyzed the clinical and pathological data of 850 CRC patients who were admitted to Qilu Hospital of Shandong University between July 2017 and June 2020 and met the inclusion criteria. The study included patients who had undergone electronic colonoscopy and were diagnosed with CRC by pathology. Patients who had received anti-tumor treatment for colorectal or other tumors before obtaining the pathological specimens, or those with a history of intestinal resection outside the appendix, were excluded from the study. The above inclusion and exclusion criteria are designed to ensure that the research subjects are newly diagnosed CRC patients who have not received anti-tumor treatment, so as to accurately reflect their natural characteristics at the initial diagnosis. The patients were divided into three age groups: <40 years, 40–50 years, and >50 years.

### Ethical considerations

This study was approved by the Ethics Committee of Qilu Hospital of Shandong University, and conducted according to the Declaration of Helsinki (Approval KYLL-202008-079). Given the retrospective nature of the study and anonymous data, the need for informed consent was waived.

### Research content

Basic information includes age, gender, comorbidities, medical history, and family history. Clinical symptoms include bloody or black stools; changes in bowel habits, abdominal pain, distension or discomfort; abdominal masses; anemia, fatigue or weight loss; rectal fullness; anal pain. Colonoscopy results include the location and number of tumors. Laboratory tests include occult blood (OB), hemoglobin (HGB), albumin (ALB); tumor markers include carcinoembryonic antigen (CEA), carbohydrate antigen 125 (CA125), carbohydrate antigen 199 (CA199), and carbohydrate antigen 724 (CA724). Pathological features include gross type, histological classification, differentiation grade and Dukes pathological stage.

In this study, comorbidities include hypertension, diabetes and coronary heart disease. Medical history includes colorectal polyps, chronic appendicitis or appendectomy, chronic biliary disease or cholecystectomy. Family history includes first-degree relatives with CRC or progressive adenomatous polyposis.

Cancers of the cecum, ascending colon, hepatic region and the anterior two-thirds of the transverse colon are classified as right-sided colon cancer, while cancers of the posterior one-third of the transverse colon, splenic region, descending colon and sigmoid colon are classified as left-sided colon cancer. In this study, total colon cancer refers to a tumor involving both the right and left colon, that is the entire colon, including cecum, ascending colon, transverse colon, descending colon and sigmoid colon.

The number of CRC endoscopic tumors refers to the number of tumor lesions observed in the colon during a colonoscopy.

All tests were collected at the time of the patient’s initial diagnosis, before any surgery or anti-tumor treatment, to reflect the disease status at the time of diagnosis. The positive test criteria refer to the cut-off values of previous studies (31–33): OB positive, HGB<115g/L, ALB<35g/L, CEA>5ng/mL, CA125>35U/mL, CA199>39U/mL and CA724>6.9U/mL.

This study adopts the Dukes pathological staging system, which is intuitive in clinical practice and provides a concise description of CRC staging. It reflects tumor invasion depth and lymph node metastasis, enabling clinicians to make rapid clinical judgments. Therefore, the Dukes staging retains clinical value in CRC prognosis stratification. Multiple studies have utilized this system (34–37), so it will not significantly affect the comparability of this study with others in the staging trends. To simplify statistical analysis, this study categorizes Dukes staging into stages A+B and stages C+D. The stages A+B represent early-stage tumors, while stages C+D indicate advanced-stage tumors. This classification allows intuitive comparison of staging differences across age groups. Although this approach may omit some details, it facilitates statistical processing and clearly demonstrates staging trends among different age groups. Dukes pathological stage criteria: In stage A, the lesion is confined to the inner layer of the intestinal wall, has not penetrated the deep muscle layer, and there is no lymph node metastasis; in stage B, the lesion invades the serosal layer or extends beyond the serosa and into extraintestinal tissues, but it can still be completely resected without lymph node metastasis; in stage C, the lesion invades the full thickness of the intestinal wall or does not invade the full thickness but is accompanied by lymph node metastasis; in stage D, the cancer is associated with distant metastasis, extensive local infiltration, or widespread lymph node metastasis, and cannot be surgically removed.

Histological classification criteria refers to WHO (38): common adenocarcinoma; special types of adenocarcinoma, including mucinous adenocarcinoma, signet ring cell carcinoma, serrated adenocarcinoma, micro papillary carcinoma, medullary carcinoma, and sieve-like comedo adenocarcinoma; rare types of adenocarcinoma, including adenosquamous carcinoma, squamous cell carcinoma, spindle cell carcinoma/sarcomatoid carcinoma, and undifferentiated carcinoma; other special types.

### Statistical analysis

To analyze the clinical and pathological characteristics of different CRC age groups, the study variables are categorized into continuous variables (age) and categorical variables (others except age). Depending on the nature of the dependent variable, continuous variables are represented by mean and standard deviation, and inter-group comparisons are conducted using the Student’s t-test (The data follows a normal distribution with homogeneous variance) or Mann Whitney U-test (The data does not follow a normal distribution with heterogeneous variance). Categorical variables are presented as frequency and percentage, and inter-group comparisons are conducted using Pearson chi-square test or Fisher’s exact test. All statistical tests are two-sided with p-value of <0.05 considered significant. Analyses are performed using IBM SPSS v.29 software (Armonk, NY, USA).

## Results

### Basic information

This study included 850 patients with CRC, ranging in age from 15 to 90 years, with an average age of 61.7 ± 12.4 years. The groups comprised 40, 108, and 702 patients, respectively, with average ages of 31.3 ± 6.1, 45.8 ± 2.9, and 65.9 ± 8.6 years. All three groups had more male patients than female patients, with a total of 489 males and 361 females, giving a male-to-female ratio of approximately 1.4:1. The male proportions in each group were 62.5%, 63.0% and 56.4% and the gender differences among the three groups were not statistically significant (p=0.355). Among patients under 40 years and 40–50 age groups, the proportions of comorbidities and medical history were lower (**Table 1**), and the opposite was true in the over 50 age group. In terms of comorbidities, the proportion of hypertension was 2.5%, 15.7% and 36.9%; the proportion of diabetes was 5.0%, 11.1% and 18.4%. Coronary heart disease was only seen in the >50 years group, with a proportion of 14.0%. Neither the < 40 years old group nor the 40–50 years old group had a history of coronary heart disease. In the group under 40 years old, only 3 patients had comorbidities, with the lowest proportion being only 7.5%. There were statistically significant differences in the three comorbidities among the three groups (p<0.001, p=0.021, p<0.001). In terms of medical history, chronic appendicitis or appendectomy was more common in the <40 age group, which was the only medical condition in this age group. The proportions of these conditions in each group were 12.5%, 7.4%, and 7.7%, respectively. In contrast, the other two conditions—colorectal polyps and chronic biliary disease or cholecystectomy—were more prevalent in the >50 age group. Except for the <40 age group, the proportions in the other two groups were 0.9% and 2.6% for colorectal polyps, and 2.8% and 6.8% for chronic biliary disease or cholecystectomy. There were no statistically significant differences in the three types of past medical histories among the three groups (p=0.528, p=0.537, p=0.074). In terms of family history, the proportion of family history was relatively high in the < 40 years old and 40–50 years old groups. Particularly, the < 40 years old group had the highest proportion at 10%, while the other two groups accounted for 9.3% and 3.8% respectively. There was a statistically significant difference in family history among the three groups (p=0.015).

**Table 1:** Basic information.

Variables	Age<40 (n=40)	Age40-50 (n=108)	Age>50 (n=702)	P-value
Sex				0.355^+^
Males	25 (62.5%)	68 (63.0%)	396 (56.4%)	
Females	15 (37.5%)	40 (37.0%)	306 (43.6%)	
Comorbidities ^a^				
Hypertension				**<0.001** ^+^
Yes	1 (2.5%)	17 (15.7%)	260 (37.0%)	
No	39 (97.5%)	91 (84.3%)	442 (63.0%)	
Diabetes				**0.021** ^+^
Yes	2 (5.0%)	12 (11.1%)	129 (18.4%)	
No	38 (95.0%)	96 (88.9%)	573 (81.6%)	
Coronary heart disease				**<0.001^++^**
Yes	0 (0.0%)	0 (0.0%)	98 (14.0%)	
No	40 (100.0%)	108 (100.0%)	604 (86.0%)	
Medical history ^b^				
Colorectal polyps				0.528**^++^**
Yes	0 (0.0%)	1 (0.9%)	18 (2.6%)	
No	40 (100.0%)	107 (99.1%)	684 (97.4%)	
Chronic appendicitis or appendectomy				0.537^+^
Yes	5 (12.5%)	8 (7.4%)	54 (7.7%)	
No	35 (87.5%)	100 (92.6%)	648 (92.3%)	
Chronic biliary disease or cholecystectomy				0.074**^++^**
Yes	0 (0.0%)	3 (2.8%)	48 (6.8%)	
No	40 (100.0%)	105 (97.2%)	654 (93.2%)	
Family history				**0.015** ^+^
Yes	4 (10.0%)	10 (9.3%)	27 (3.8%)	
No	36 (90.0%)	98 (90.7%)	675 (96.2%)	

^a b^: One person may have one or more comorbidities or medical history; p^+^:Pearson chi-square test; p^++^: Fisher’s exact test. Bold p-values indicate statistically significant differences (p<0.05).

### Clinical symptoms

**Figure 1:**
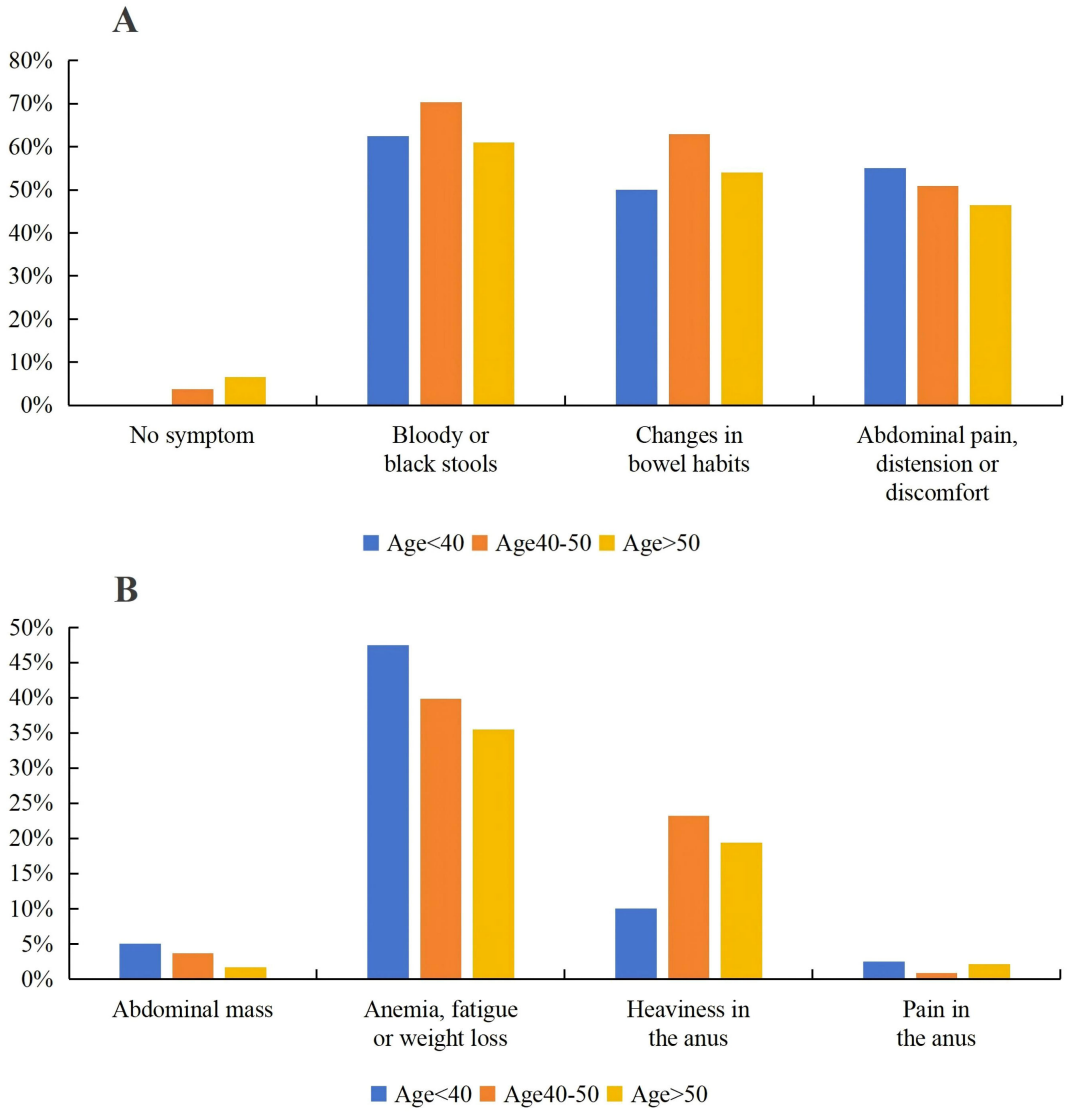
The proportion of clinical symptoms under different age groups. **(A)** Main symptoms and no symptom: bloody or black stools; changes in bowel habits; abdominal pain, distension or discomfort; **(B)** Secondary symptoms: abdominal mass; anemia, fatigue or weight loss; heaviness in the anus; pain in the anus.

In this study, patients under 40 years old and those aged 40–50 years old presented with more symptoms, especially those under 40 years old all showed symptoms, and the clinical manifestations were diverse. In contrast, there were 3.7% and 6.6% of patients symptom-free in the other two groups, respectively (**Figure 1A**). There was no statistically significant difference in symptom distribution among the three groups (p=0.161). The most common symptoms in the three groups were as follows: bloody or black stools, changes in bowel habits, and abdominal pain, distension or discomfort. The proportions of these symptoms in each group were: bloody or black stools at 62.5%, 70.4% and 61.0%; changes in bowel habits at 50.0%, 63.0% and 54.1%; and abdominal pain, distension, or discomfort at 55.0%, 50.9% and 46.4%. Other common symptoms included anemia, fatigue or weight loss, and rectal fullness (**Figure 1B**). The proportions for these symptoms by age group were: anemia, fatigue or weight loss at 47.5%, 39.8% and 35.5%; and rectal fullness at 10.0%, 23.2% and 19.4%. Less common symptoms included abdominal masses and anal pain, with proportions of 5.0%, 3.7% and 1.7% for abdominal masses; and 2.5%,0.9% and 2.1% for anal pain. Among them, the <40 years group had higher proportions of abdominal pain, distension or discomfort; abdominal masses; anemia, fatigue or weight loss, and anal pain. The 40–50 years group had higher proportions of changes in bowel habits, bloody or black stools, and rectal fullness. The >50 years group did not show significant differences in various symptoms (**Supplementary Table 1**).

### Colonoscopy results

The primary disease site in all three groups was rectum, with the proportions being 52.5%, 48.1% and 52.4%, respectively (**Table 2**). In the <40 age group, left-sided colon tumors were more common than in other groups, with proportions of 37.5%, 25.0% and 23.1%, respectively. In the 40–50 age group, right-sided colon tumors were more common, with proportions of 10.0%, 25.9% and 21.9%, respectively.In the >50 age group, tumors were more commonly found in the entire colon and entire rectum, with the entire colon being present only in the >50 age group at a rate of 0.6% and the entire rectum only being present in the 40–50 and >50 age groups at rates of 0.9% and 2.0%, respectively. All three groups predominantly had solitary tumors. In the <40 age group, all patients had single tumors. A small number of patients in the other two groups had multiple tumors, with 3 (2.8%) patients in the 40–50 age group and 30 (4.3%) patients in the >50 age group. There was no statistically significant difference in tumor location among the three groups (p=0.392), but there was a statistically significant difference in the number of tumors (p=0.012).

**Table 2:** Colonoscopy results.

Variables	Age<40 (n=40)	Age40-50 (n=108)	Age>50 (n=702)	P-value
Tumor site				0.392**^++^**
Right-side colon	4 (10.0%)	28 (25.9%)	154 (21.9%)	
Left-side colon	15 (37.5%)	27 (25.0%)	162 (23.1%)	
Total colon	0 (0.0%)	0 (0.0%)	4 (0.6%)	
Rectum	21 (52.5%)	52 (48.1%)	368 (52.4%)	
Colorectum	0 (0.0%)	1 (0.9%)	14 (2.0%)	
Number of tumors				**0.012^++^**
1	40 (100%)	105 (97.2%)	672 (95.7%)	
≥2	0 (0.0%)	3 (2.8%)	30 (4.3%)	

p^++^: Fisher’s exact test. Bold p-values indicate statistically significant differences (p<0.05).

### Laboratory test results

In this study, the highest positive rates for all three groups were for OB. The positive rates for HGB and ALB in the <40 age group were higher than other indicators, with proportions of OB being 86.2%, 87.3% and 85.1%; HGB being 50.0%, 38.9% and 35.7%; and ALB being 32.5%, 21.3% and 32.0% in each group, respectively (**Table 3**). The differences in OB, HGB, and ALB levels among the three groups were not statistically significant (p=0.881, p=0.169, p=0.078). CA125 and CA724 had significantly higher positive rates in the <40 age group, while CA199 had a higher positive rate in the 40–50 age group, and CEA had a higher positive rate in the>50 age group. The proportions of the above tumor markers in each group were: 31.6%, 11.0% and 9.4% for CA125; 48.4%, 26.7% and 23.1% for CA724; 21.0%, 23.5% and 18.5% for CA199; and 4.2%, 39.2% and 43.2% for CEA. Among these, the tumor marker with the highest positive rate in the <40 age group was CA724, while CEA had the highest positive rate in both the 40–50 and >50 age groups. The differences in CA125 and CA724 levels among the three groups were statistically significant (p<0.01), while the differences in CEA and CA199 levels were not statistically significant (p=0.441, p=0.463).

**Table 3:** Laboratory tests results.

Laboratory tests	Age<40 (n=40)	Age40-50 (n=108)	Age>50 (n=702)	P-value
OB				0.881^+^
Positive	25 (86.2%)	62 (87.3%)	424 (85.1%)	
Negative	4 (13.8%)	9 (12.7%)	74 (14.9%)	
HGB				0.169^+^
Positive	20 (50.0%)	42 (38.9%)	250 (35.7%)	
Negative	20 (50.0%)	66 (61.1%)	450 (64.3%)	
ALB				0.078^+^
Positive	13 (32.5%)	23 (21.3%)	224 (32.0%)	
Negative	27 (67.5%)	85 (78.7%)	476 (68.0%)	
CEA				0.441^+^
Positive	13 (34.2%)	40 (39.2%)	292 (43.2%)	
Negative	25 (65.8%)	62 (60.8%)	384 (56.8%)	
CA125				**<0.01** ^+^
Positive	12 (31.6%)	11 (11.0%)	62 (9.4%)	
Negative	26 (68.4%)	89 (89.0%)	596 (90.6%)	
CA199				0.463^+^
Positive	8 (21.0%)	24 (23.5%)	124 (18.5%)	
Negative	30 (79.0%)	78 (76.5%)	547 (81.5%)	
CA724				**<0.01** ^+^
Positive	15 (48.4%)	24 (26.7%)	140 (23.1%)	
Negative	16 (51.6%)	66 (73.3%)	467 (76.9%)	

OB, occult blood in stool; HGB, hemoglobin; ALB, albumin; CEA, carcinoembryonic antigen; CA125, carbohydrate antigen 125; CA199, carbohydrate antigen 199; CA724, carbohydrate antigen 724; p^+^, Pearson chi-square test. Bold p-values indicate statistically significant differences (p<0.05).

### Pathological features

The pathological characteristics of the three groups were primarily ulcerative, moderately differentiated and adenocarcinoma types, with the proportions being 40.0%, 54.6% and 55.4% for ulcerative type; 37.5%, 50.0% and 53.6% for moderately differentiated type; and 72.5%, 75.9% and 83.0% for adenocarcinoma type in each group, respectively (**Table 4**). In terms of gross type, the cauliflower, exophytic, and undetermined types were more prevalent in the <40 age group, with proportions of 5.0%, 2.8% and 1.6% for cauliflower type; 2.5%, 0.9% and 1.6% for exophytic type; and 37.5%, 22.2% and 24.1% for undetermined type in each group, respectively. The protruding and fungating types were more prevalent in the 40–50 age group, with proportions of 15.0%, 18.5% and 17.7% for protruding type; and 0%, 0.9%, and 0.6% for fungating type in each group, respectively. The ulcerative type was more prevalent in patients over 50 years old, with proportions of 40.0%, 54.6% and 55.4% in each group, respectively. In terms of histological classification, mucinous adenocarcinoma and signet ring cell carcinoma were more prevalent in the <40 years group, with proportions of 15.0%, 9.3% and 3.6% for mucinous adenocarcinoma; and 5.0%, 0.9% and 0.3% for signet ring cell carcinoma in each group, respectively. Only the >50 years group had squamous cell carcinoma and medullary carcinoma, with proportions of 0.3% and 0.1%, respectively; while the other two groups did not have these types. In terms of differentiation grade, Poor differentiation and well differentiation were more common in the <40 years group, with proportions of 7.5%, 5.6% and 5.4% for poor differentiation; and 7.5%, 3.7% and 5.3% for well differentiation in each group, respectively. Moderate differentiation were more common in the >50 years group, with proportions of 37.5%, 50.0% and 53.6% in each group, respectively. In terms of Dukes pathological stage, the <40 years group mainly had D stage tumors, the 40–50 years group mainly had B stage tumors, and the>50 years group mainly had C stage tumors (**Table 5**). The proportions for each group were 45.0%, 24.1% and 15.5% for D stage; 25.0%, 30.6% and 27.5% for B stage; and 20.0%, 27.8% and 31.1% for C stage. In summary, the <40 and 40–50 years group predominantly had late-stage tumors (C+D stage), with proportions of 65.0%, 51.9% and 46.6% in each group, respectively. In contrast, the >50 years group predominantly had early-stage tumors (A+B stage), with proportions of 35.0%, 48.2% and 53.4% in each group, respectively (**Supplementary Table 2**). There were statistically significant differences in histological classification and Dukes pathological stage among the three groups (p=0.002, p<0.01), while there were no statistically significant differences in gross type and differentiation grade (p=0.332, p=0.086).

**Table 4:** Pathological features.

Variables	Age<40 (n=40)	Age40-50 (n=108)	Age>50 (n=702)	P-value
Gross type				0.332^++^	
Ulcerative type	16 (40.0%)	59 (54.6%)	389 (55.4%)		
Protruding type	6 (15.0%)	20 (18.5%)	124 (17.7%)		
Cauliflower type	2 (5.0%)	3 (2.8%)	11 (1.6%)		
Fungating type	0 (0.0%)	1 (0.9%)	4 (0.6%)		
Exophytic type	1 (2.5%)	1 (0.9%)	11 (1.6%)		
Undetermined	15 (37.5%)	24 (22.2%)	163 (23.1%)		
Histological classification				**0.002** ^++^	
Adenocarcinoma	29 (72.5%)	82 (75.9%)	583 (83.0%)		
Mucinous adenocarcinoma	6 (15.0%)	10 (9.3%)	25 (3.6%)		
Signet ring cell cancer	2 (5.0%)	1 (0.9%)	2 (0.3%)		
Squamous cell carcinoma	0 (0.0%)	0 (0.0%)	2 (0.3%)		
Medullary carcinoma	0 (0.0%)	0 (0.0%)	1 (0.1%)		
Mixed	3 (7.5%)	15 (13.9%)	89 (12.7%)		
Differentiation grade				0.086^++^	
Poor differentiation	3 (7.5%)	6 (5.6%)	38 (5.4%)		
Moderate differentiation	15 (37.5%)	54 (50.0%)	376 (53.6%)		
Well differentiation	3 (7.5%)	4 (3.7%)	37 (5.3%)		
Moderate-to-poor differentiation	3 (7.5%)	14 (13.0%)	83 (11.8%)		
Moderate-to-well differentiation	1 (2.5%)	11 (10.2%)	63 (9.0%)		
Undetermined	15 (37.5%)	19 (17.6%)	105 (14.9%)		

p^++^: Fisher’s exact test. Bold p-values indicate statistically significant differences (p<0.05).

**Table 5:** Dukes pathological stage.

Stages	Age<40 (n=40)	Age40-50 (n=108)	Age>50 (n=702)	P-value
Stage A	4 (10.0%)	19 (17.6%)	182 (25.9%)	**<0.01** ^+^
Stage B	10 (25.0%)	33 (30.6%)	193 (27.5%)	
Stage C	8 (20.0%)	30 (27.8%)	218 (31.1%)	
Stage D	18 (45.0%)	26 (24.1%)	109 (15.5%)	

p^+^:Pearson chi-square test. Bold p-value indicates statistically significant differences (p<0.05).

## Discussion

Our research indicated that CRC patients at different age levels showed varying clinical manifestations and pathological features. Our study showed that a higher proportion of the patients included were male, and the average age was 61.7 years. This was consistent with the findings of Sung et al. (39), who found that CRC incidence peaked in people aged 50 to 75 and that men were at higher risk. This gender difference may be linked to dietary, lifestyle (40) and the preventive effects of estrogen (41). In our study, the incidence of both comorbidities and medical history was higher in the >50 years group, which aligned with the trend of increased chronic disease burden among the elderly population. Specifically, the detection rates of common chronic diseases such as hypertension, diabetes, and coronary heart disease in patients over 50 years old are significantly higher than those in younger patients. This may be related to the decline in physical functions and metabolic capacity among the elderly. This suggests that in clinical work, more attention should be paid to the management of complications in LOCRC patients to formulate more comprehensive and personalized treatment plans. We also found that the proportion of family history in patients under 40 years old was higher, indicating a higher genetic susceptibility to cancer in EOCRC patients. This finding is consistent with previous studies (42), suggesting that in the clinical diagnosis and treatment process, more attention should be paid to genetic screening and family history inquiries for young CRC patients, so as to identify high-risk groups at an early stage and take corresponding preventive measures.

Clinical symptoms in CRC varied across different age groups. We found that patients under 40 years old had a wide range of symptoms, which may be related to their higher susceptibility to anxiety and faster tumor progression (43). Bloody or black stools, changes in bowel habits, and abdominal pain, distension or discomfort were common across all age groups, with bloody or black stools being the most prevalent, confirming that bleeding is a primary sign of CRC in all age groups (44). Additionally, patients under 40 were more likely to present with abdominal masses and anemia, fatigue or weight loss; while those aged 40–50 were more likely to experience changes in bowel habits, bloody or black stools. These differences may be attributed to factors such as the tumor location, tumor size and extent of tumor invasion, suggesting the need for more accurate early warning signs of CRC (45).

However, despite the diverse clinical manifestations of young patients, there was no statistically significant difference in clinical symptoms among different age groups (p=0.161), indicating that it is difficult to reliably distinguish early CRC solely based on clinical manifestations. Previous studies have shown that although EOCRC patients show different symptoms, they often ignore them (46). While CRC patients over 50 typically have less noticeable clinical symptoms which are often discovered during physical exams, which may lead to delayed diagnosis of the disease (47). Therefore, young people with symptoms should be given due attention. Early screening, diagnosis and treatment of CRC should be carried out as soon as possible to avoid missed diagnosis and misdiagnosis.

In our study, the rectum was the most common site of CRC across all age groups, particularly in those under 40 years old. Additionally, the incidence of left-sided colon cancer was higher in the <40 age group; whereas, the incidence of right-sided colon cancer was relatively higher in the 40–50 age group. These differences may be attributed to variations in intestinal anatomy, physiological functions, and the intestinal microenvironment (17). Although younger patients appeared to have more left-sided tumors, there was no statistically significant difference in tumor location (p=0.392). This trend warrants exploration in larger studies. For total CRC, the incidence was higher in patients over 50 years old, possibly due to the cumulative effects of intestinal lesions in middle-aged and older adults. In terms of tumor numbers, single tumor was predominant across all age groups, especially in patients under 40, where all cases were single tumor. As age increased, the proportion of multiple tumors gradually increased. This may reflect differences in the progression rate and invasiveness of intestinal lesions among patients of different ages.

The highest positive rates for laboratory tests across all age groups were OB in our study. Young patients are prone to anemia and hypoproteinemia, which may be due to their vigorous metabolism, high nutritional demands, and unhealthy lifestyles such as unbalanced diet and overwork. At the same time, the disease itself may affect intestinal absorption function, leading to absorption disorders of nutrients such as iron and protein, and promoting the occurrence of anemia and hypoproteinemia. However, there was no statistically significant difference in the results of OB, HGB, and ALB (p=0.881, p=0.169, p=0.078), possibly because the results of them were easily influenced by factors such as nutritional status and chronic diseases, making them unsuitable as standalone indicators for assessing the severity of CRC (48). Therefore, in clinical practice, it is necessary to comprehensively evaluate the severity of CRC by combining other more sensitive tumor markers and imaging examinations, etc., in order to improve the accuracy of diagnosis and the effectiveness of treatment.

Regarding tumor marker results, patients under 40 years old have a higher positive rate for CA125 and CA724, while the 40–50 and over 50 years old groups have the highest positive rate for CEA. Elevated CA125 may be related to the tendency of peritoneal metastasis. Even if there is no obvious metastasis, the risk of micrometastasis is higher in younger patients. CA724 is a high-molecular-weight glycoprotein. Its elevation may be associated with stronger immune escape and interstitial infiltration characteristics in young CRC tumors. Young patients have a high proportion of mucinous adenocarcinoma and signe-ring cell carcinoma, which are rich in glycosylated antigens and thereby stimulate the secretion of CA724. This suggests that for patients under 40 years old, although tumor markers such as CA724 and CA125 lack specificity, combined detection can improve the sensitivity of diagnosis. As a broad-spectrum tumor marker, the positive rate of CEA in patients over 40 years old was higher, which may be related to its role in tumor progression (49). As the age of 40-year-old CRC patients increases, the tumor may be at a relatively later stage, with more active proliferation of tumor cells and a greater tumor burden. This may prompt an increase in CEA secretion, thereby leading to a higher positive rate. Therefore, in clinical practice, for CRC patients over 40 years old, the test results of CEA have significant reference value in evaluating tumor progression, formulating treatment plans, and monitoring prognosis.

Adenocarcinoma was the most common histological classification in our study across all age groups. However, there were variations in the distribution of specific types of adenocarcinoma, such as mucinous adenocarcinoma and signet ring cell carcinoma, which were more prevalent in the <40 age group. These specific types of adenocarcinoma often show stronger invasiveness and poorer prognosis. This finding is consistent with the results of Rho et al. (18), who compared compared CRC in the younger group (18–44 years) and the older group (≥45 years), finding that despite similar treatment patterns and survival outcomes to the older group, CRC patients in the younger group showed more aggressive biological behavior. In addition, in this study, all three groups of patients were mainly ulcerative and moderately differentiated. Poorly differentiated patients were more common in the age group <40 years old, while moderately differentiated patients were more prevalent in the age group >50 years old. However, there were no statistically significant differences among the three groups in terms of gross classification and degree of differentiation (p=0.332, p=0.086). Although no significant difference was statistically observed, this trend still suggests that there may be some potential association between age and the degree of tumor differentiation. Poorly differentiated tumor cells often have stronger proliferation and invasion capabilities, which may partly explain why young patients, although their overall survival outcomes are similar to those of elderly patients, exhibit more aggressive biological behaviors. In the future, studies with larger sample sizes will be needed to further verify this hypothesis and deeply explore the molecular biological mechanisms behind it. It should be noted that in both of these research factors, a certain proportion of patients showed undetermined pathological results, especially those under 40 years old. The possible reason lies in that for some young patients, due to the late discovery of the disease, the tumor tissue has already undergone necrosis or fibrosis, making it difficult to obtain pathological samples. In cases where tumor heterogeneity is strong or biopsy specimens are limited, it is difficult to accurately grade some tumors with atypical or mixed differentiation features. Therefore, this suggests that in the process of clinical pathological diagnosis, especially for young patients with CRC, the influence of the tumor tissue state and the limitations of biopsy specimens on the pathological results should be fully considered. When necessary, multiple biopsies can be performed or the resection depth can be increased to obtain sufficient tissue, thereby improving the accuracy of pathological assessment and the rate of disease diagnosis.

Previous studies have shown that EOCRC patients are often diagnosed with late-stage tumors, and our study confirmed this. In the >50 age group, early-stage tumors (A+B stages) were more common, while in the <50 age group, late-stage tumors (C+D stages) were more prevalent. The differences in pathological stage among the three groups were statistically significant (p<0.01). As Willauer et al. (50) found that EOCRC was associated with aggressive biological behavior, including a higher incidence of mucinous adenocarcinoma or signet ring cell carcinoma. This suggests that EOCRC tumors have stronger invasiveness and metastatic potential, and are diagnosed at a relatively late-stage stage (51, 52). In contrast, LOCRC patients who have more screening opportunities, often detect tumors at an early stage, but a significant number still remain at a late-stage stage, indicating the need for enhanced screening of LOCRC to improve the detection rate of CRC at an early stage. In view of the special pathological characteristics of young patients, clinical treatment strategies should also be adjusted accordingly, such as considering more aggressive surgical plans and postoperative adjuvant therapy, with the aim of improving the prognosis of patients.

Previous studies have typically categorized CRC patients into two groups: EOCRC and LOCRC. This study further refines the age stratification, dividing patients into three age groups: <40 years, 40–50 years, and >50 years. For the increasingly concerning EOCRC patients, it allows for a deeper analysis of their characteristics in the <40 age group, providing a more detailed basis for early screening, diagnosis, and treatment strategies for this population. Additionally, most current CRC research focuses on LOCRC, with an emphasis on treatment. This study analyzes the clinical and pathological features of CRC across different age groups, highlighting the unique aspects of EOCRC. This provides strong support for addressing the trend of younger CRC onset and advancing screening ages.

In summary, this study highlighted the differences in clinical manifestations and pathological features among CRC patients under age stratification. EOCRC patients, especially those under 40 years old, have a low proportion of comorbidities and a high proportion of family history. The clinical symptoms are significant but lack specificity. Left-sided colon cancer accounts for a relatively high proportion and is mostly a single tumor. Anemia and hypoproteinemia are prone to occur, and the results of multiple tumor markers are high but lack specificity. The degree of tumor differentiation is low, with a high proportion of mucinous adenocarcinoma and signe-ring cell carcinoma. The pathological stage is mostly advanced, and the prognosis is usually poor. Therefore, clinicians should remain highly vigilant for young people with persistent digestive tract symptoms and actively recommend that they undergo early colonoscopy. By comprehensively evaluating multiple test indicators, potential CRC lesions can be detected as early as possible to avoid missed diagnosis or misdiagnosis. For CRC patients under the age of 40, especially those with a family history, it is recommended that they undergo genetic testing and genetic counseling as early as possible to achieve early intervention. In the formulation of treatment plans, the pathological characteristics of EOCRC patients, such as tumor location, pathological type and stage, should be fully considered to develop individualized treatment strategies, with the aim of improving the prognosis of patients.

## Data Availability

The original contributions presented in the study are included in the article/**Supplementary Material**. Further inquiries can be directed to the corresponding author.

## Supplementary material

Supplementary Table 1 Clinical symptoms ^a^. ^a^: One person may have one or more clinical symptoms; p^+^:Pearson chi-square test; p^++^: Fisher’s exact test.

Supplementary Table 2 Clinical symptoms Dukes pathological stage (classification). p^+^:Pearson chi-square test.
