# Supplementary material for: Clinical and pathological characteristics of patients with colorectal cancer under age stratification
Source: Front Oncol. 2026 Jan 12;15:1656277. doi: 10.3389/fonc.2025.1656277 (PMC12832363; doi:10.3389/fonc.2025.1656277)
Supplement: Supplementary Table 1 — Clinical symptoms a. a: One person may have one or more clinical symptoms; p+:Pearson chi-square test; p++: Fisher’s exact test. [file Table1.docx]

**Supplementary Table 1 : Clinical symptoms** ^a^

| Symptoms | Age<40  (n=40) | Age40-50  (n=108) | Age>50  (n=702) | p-value | |  |
| --- | --- | --- | --- | --- | --- | --- |
| No symptom |  |  |  | | 0.161^++^ | |
| Yes | 0 (0.0%) | 4 (3.7%) | 46 (6.6%) | |  | |
| No | 40 (100.0%) | 104 (96.3%) | 656 (93.4%) | |  | |
| Bloody or black stools |  |  |  | | 0.172^+^ | |
| Yes | 25 (62.5%) | 76 (70.4%) | 428 (61.0%) | |  | |
| No | 15 (37.5%) | 32 (29.6%) | 274 (39.0%) | |  | |
| Changes in bowel habits |  |  |  | | 0.184^+^ | |
| Yes | 20 (50.0%) | 68 (63.0%) | 380 (54.1%) | |  | |
| No | 20 (50.0%) | 40 (37.0%) | 322 (45.9%) | |  | |
| Abdominal pain, distension or discomfort |  |  |  | | 0.422^+^ | |
| Yes | 22 (55.0%) | 55 (50.9%) | 326 (46.4%) | |  | |
| No | 18 (45.0%) | 53 (49.1%) | 376 (53.6%) | |  | |
| Abdominal masses |  |  |  | | 0.104^++^ | |
| Yes | 2 (5.0%) | 4 (3.7%) | 12 (1.7%) | |  | |
| No | 38 (95.0%) | 104 (96.3%) | 690 (98.3%) | |  | |
| Anemia, fatigue or weight loss |  |  |  | | 0.233^+^ | |
| Yes | 19 (47.5%) | 43 (39.8%) | 249 (35.5%) | |  | |
| No | 21 (52.5%) | 65 (60.2%) | 453 (64.5%) | |  | |
| Rectal fullness |  |  |  | | 0.199^+^ | |
| Yes | 4 (10.0%) | 25 (23.2%) | 136 (19.4%) | |  | |
| No | 36 (90.0%) | 83 (76.8%) | 566 (80.6%) | |  | |
| Anal pain |  |  |  | | 0.570^++^ | |
| Yes | 1 (2.5%) | 1 (0.9%) | 15 (2.1%) | |  | |
| No | 39 (97.5%) | 107 (99.1%) | 687 (97.7%) | |  | |
|  |  |  |  | |  | |

^a^ : One person may have one or more clinical symptoms. p^+^:Pearson chi-square test; p^++^: Fisher's exact test.

**Supplementary Table 2 : Clinical symptoms Dukes pathological stage (classification)**

| Stages | Age<40  (n=40) | Age40-50  (n=108) | Age>50  (n=702) | p-value |
| --- | --- | --- | --- | --- |
| Stage A+B | 14 (35.0%) | 52 (48.2%) | 375 (53.4%) | 0.054^+^ |
| Stage C+D | 26 (65.0%) | 56 (51.9%) | 327 (46.6%) |  |

p^+^:Pearson chi-square test.
